# *Agaricus brasiliensis* polysaccharides stimulate human monocytes to capture *Candida albicans,* express toll-like receptors 2 and 4, and produce pro-inflammatory cytokines

**DOI:** 10.1186/s40409-017-0102-2

**Published:** 2017-03-23

**Authors:** Priscila Raquel Martins, Ângela Maria Victoriano de Campos Soares, Andrea Vanessa da Silva Pinto Domeneghini, Márjorie Assis Golim, Ramon Kaneno

**Affiliations:** 1 Department of Microbiology and Immunology, Botucatu Biosciences Institute, São Paulo State University (UNESP – Univ Estadual Paulista), Botucatu, SP Brazil; 2 Department of Pathology, Botucatu Medical School, São Paulo State University (UNESP – Univ Estadual Paulista), Botucatu, SP Brazil; 3 Department of Pathology, Paulista Central University Center (UNICEP), São Carlos, SP Brazil; 4 Blood Bank Division, Botucatu Medical School, São Paulo State University (UNESP – Univ Estadual Paulista), Botucatu, SP Brazil

**Keywords:** *Agaricus blazei*, *Agaricus brasiliensis*, Monocytes, Pattern recognition receptors (PPR), Toll-like receptors

## Abstract

**Background:**

*Agaricus brasiliensis* is a medicinal mushroom with immunomodulatory and antitumor activities attributed to the β-glucans presented in the polysaccharide fraction of its fruiting body. Since β-glucans enhance cellular immunoresponsiveness, in this study we aimed to evaluate the effect of an acid-treated polysaccharide-rich fraction (ATF) of *A. brasiliensis* on the ability of human monocytes to adhere/phagocyte *C. albicans* yeast cells, their expression of pattern recognition receptors and their ability to produce cytokines.

**Methods:**

Adhesion/phagocytosis of FITC-labeled *C. albicans* was evaluated by flow cytometry. Cells were incubated with specific fluorochrome-labeled antibodies for TLR2 and 4, βGR and MR and also evaluated by flow cytometry. Monocytes were cultured with ATF, and culture supernatants were collected for analysis of in vitro cytokine production by ELISA (TNF-α, IL-1β, IL-12 and IL-10).

**Results:**

ATF significantly increased the adherence/phagocytosis of *C. albicans* by monocytes and this was associated with enhanced expression of TLR2 and TLR4, while no effect was observed on βGR or MR. Moreover, expression of TLR4 and TLR2 was associated with higher levels of in vitro production of TNF-α and IL-1, respectively. Production of IL-10 was also increased by ATF treatment, but we found no association between its production and the expression of Toll-like receptors.

**Conclusion:**

Our results provided us with evidence that *A. brasiliensis* polysaccharides affect human monocytes probably through the modulation of Toll-like receptors.

## Background

*Agaricus brasiliensis* Wasser et al. (formerly *Agaricus blazei* Murrill) is a mushroom native to Brazil that has a wide range of medicinal properties including antitumor and immunostimulatory activities [1–6]. These activities are mainly attributed to β-glucans found in the polysaccharide fraction of their fruiting bodies [3, 5, 7]. It was previously reported that the acid-treated fraction (ATF) is polysaccharide rich and contains a large amount of β-glucans [1, 5]. These polysaccharides have the ability to cause tumor infiltration by NK cells, inhibition of in vitro tumor cell growth, and induction of apoptosis in tumor cells [5, 8]. Sorimachi et al. [9] observed that extracts of *A. blazei* can activate macrophage functions whereas our group found that products of this mushroom inhibit the growth of Ehrlich tumor both by restoring NK activity and modulating the cytokine production by spleen cells [1, 10].

Although anti-infectious properties of this mushroom have been poorly explored, it was reported that *Leishmania* species are sensitive to *Agaricus* compounds. Moreover, these subtances are able to improve the resistance of mice to experimental infection with this protozoa [11, 12]. It was also described that polysaccharides and sulfated extract can inhibit the cytolytic activity of virus such as herpes virus HSV-1 and −2; however, no direct virucidal activity had been observed [13, 14].

We have previously observed that the acid-treated polysaccharide fraction of *A. brasiliensis* (ATF) can increase the resistance of mice against *C. albicans* by stimulating fungicidal activity of peritoneal macrophages, characterized by increased levels of H_2_O_2_, and increased expression of mannose receptor [15]. Then, we asked whether human monocytes would also be stimulated by *A. brasiliensis* β-glucan. Since activation of innate immunity and stimulation of proinflammatory cytokines involve recognition of microbial pathogen-associated molecular patterns (PAMPs) by the pattern recognition receptors (PRRs), we also asked whether the putative effect on monocyte activities would be associated with stimulation of these receptors [16].

Toll-like receptors (TLRs) belong to a family of receptors that react with foreign molecules both on cell surface and inside the cytoplasmic vesicles, hence the main receptors for fungal PAMPs are TLR2/1, TLR4 and TLR2/6 expressed on cell surface [17]. Mannose receptor (MR) recognizes the mannan component of yeast cell walls, while dectin-1 is the major receptor for fungal 1–3 β-glucans involved in the phagocytosis of microorganisms together with TLRs by murine cells [18–22]. The human homologue of dectin-1 is the β-glucan receptor (βGR), with two major isoforms βGRA and βGRB [23]. Given the importance of this variety of receptors in the recognition of pathogens and activation of phagocytes, we hypothesized that administration of immunomodulatory agents, such as mushroom polysaccharides, could stimulate their expression for improving the treatment of infectious diseases.

In order to test such hypothesis and based on our previous results, in the present study we evaluated the stimulatory effect of a polysaccharide rich fraction of *A. brasiliensis* on human monocytes. We observed that ATF significantly increased the adherence/phagocytosis of *C. albicans* by monocytes by modulating the expression of TLR2 and TLR4 and the production of TNF-α and IL-1β.

## Methods

### Human peripheral monocytes

The subjects of this study included healthy blood donors from the University Hospital of the Botucatu Medical School (UNESP), Brazil, with ages ranging from 25 to 50 years. The investigation was carried out according to the Helsinki’s Declaration and approved by the institutional Research Ethics Committee. All the cell donors were informed about the procedures, objectives and risks involved in the study, and signed an agreement form (OF. 17/2007-CEP).

Peripheral blood mononuclear cells (PBMCs) were isolated from heparinized peripheral blood of seven healthy adult donors by centrifugation on Histopaque®-1077 gradient (Sigma-Aldrich Inc., USA). Cell suspension containing PBMC was washed twice with RPMI 1640 tissue culture medium (Sigma-Aldrich) and suspended in a complete culture medium [RPMI 1640 supplemented with 2 mM L-glutamine (Sigma-Aldrich), 40 μg.mL^−1^ gentamicin (Gibco Laboratories, USA) and 10% heat-inactivated autologous human serum]. Monocytes were identified by neutral red staining, and cell suspension was adjusted to 10^6^ monocytes/mL for the assays.

### Acid-treated polysaccharide fraction of *A. brasiliensis* (ATF)

The polysaccharide-rich acid-treated fraction (ATF) of *A. brasiliensis* was obtained according to the method described by Fujimyia et al. [5] and slightly modified by Pinto et al. [1]. Briefly, 800 g of a dry powdered sample of *A. brasiliensis* was mixed with 80% ethanol and boiled for 15 h in a closed system. After this period, the supernatant (ethanolic fraction) was discarded and the process was repeated twice. After the last extraction with ethanol, the pellet was mixed with distilled water, boiled for 15 h (three times) and then mixed with 5% ammonium oxalate and extracted twice at the boiling point for 10 h.

The supernatants were pooled and filtered to remove insoluble particles (Millipore cod. 2502500) followed by dialysis for 72 h against distilled water. The efficiency of dialysis for the removal of residual ammonium was checked by analysis of dialysis liquid with Nessler’s solution. The presence of oxalate was verified by heating ATF samples with 500 μL of 0.1 N potassium permanganate for 1 min. at 100 °C. The final solution of ATF was compared with a standard curve (4.0; 2.0; 0.5; 0.25 and 0.125%) prepared with 1 N HCl, and it was verified that the residual oxalate concentration was lower than 0.125%. Analysis of endotoxin contends by a *Lymulus amebocyte* lysate test (E-toxate kit – Sigma ET0200) detected less than 0.06 EU/mL.

The oxalate-soluble solution was acidified with 1 M HCl for 24 h at room temperature, followed by neutralization with 1 M NaOH (final pH = 7.0); the final solution (ATF) was lyophilized and stored at −20 °C. Before use, ATF was rehydrated with PBS and the sample was autoclaved to obtain a sterile solution. The solution was prepared by dilution of this sterile sample in complete culture medium at 500 μg/mL.

### Preparation of FITC-labeled *C. albicans*

*C. albicans* yeast cells – sample H-428/03, originally isolated from a patient of the University Hospital of Botucatu Medical School (UNESP) and maintained at −70 °C – were defrosted and grown in Sabouraud-Dextrose-Agar medium (Oxoid Ltd.) at 35 °C for 24 h. Cells were collected and washed with sterile pyrogen-free salt-solution and resuspended at 5 × 10^6^ yeast cells/mL. Viability of yeast cells was evaluated by phase-contrast microscopy (>95% viable cells).

Yeast cells were labeled with fluorescein isothiocyanate (FITC) according to Chaka et al. [24] and Szolnoky et al. [25] with slight modifications. Briefly, yeast cells were incubated with FITC (100 μg/mL) (Sigma Chemical Co.) in 0.1 M carbonate-bicarbonate buffer (pH 9.0) for 30 min. at 37 °C with occasional agitation. Yeast suspension was centrifuged for 10 min. (1000 *× g*), the supernatant was disposed and cells were washed twice with PBS (2000 *× g*). Then, yeast cells were suspended in 10 mL of 0.1 M carbonate-bicarbonate buffer containing 4% bovine serum albumin, incubated at 37 °C for 15 min., and washed twice in PBS to remove BSA-bounded FITC. Labeled yeast cells were suspended at 5 × 10^6^ cells/mL in fresh RPMI-1640.

### Adherence/phagocytosis of *C. albicans*

For evaluation of adherence/phagocytosis of yeast cells, 500 μL of PBMC suspension (10^6^ monocytes/mL) was distributed into polystyrene tubes (BD Labware) followed by incubation with 5.0 μg or 50 μg of ATF for 6 h, 12 h or 18 h at 37 °C. After incubation, cells were washed and challenged with *C. albicans*-FITC (yeast:monocyte ratio = 5:1) for 30 min. at 37 °C under a constant tension of 5% CO_2_. In order to distinguish the interaction of yeast cells with monocytes from other cells, the suspension of PBMC + *C. albicans-*FITC was incubated with anti-CD14 PerCP-Cy^TM^5.5-labeled monoclonal antibody (mAb) (BD-Pharmingen) for 20 min., at 4 °C, in a dark box. Adherence and/or phagocytosis of *C. albicans*-FITC by CD14+ cells was analyzed in a FACSCalibur flow cytometer (10,000 events per sample; Becton Dickinson) and data were analyzed by the software Cell Quest 3.1.

### Expression of surface receptors (βGR, MR, TLR2 and TLR4)

Peripheral blood mononuclear cells, containing 10^6^ monocytes/mL, were distributed (500 μL) into polystyrene tubes (BD Labware) followed by incubation with culture medium alone or culture medium plus 50 μg ATF for 6 h at 37 °C under 5% CO_2_. Cells were washed and labeled with anti-CD14 PerCP-Cy^TM^5.5 mAb (BD-Pharmingen) and mAbs for each receptor: anti-MR-FITC (BioLegend), anti-TLR2-FITC (BioLegend), and anti-TLR4-PE (BioLegend), according to the instructions of the manufacturers. Expression of β-glucans receptor (βGR) was evaluated by incubation with the primary mAb – GE2 – generously provided by Dr. Gordon Brown, Institute of Infectious Disease and Molecular Medicine, University of Cape Town, South Africa, that recognizes both βGRA and βGRB isoforms of this receptor followed by incubation with a FITC-labeled secondary antibody (Biolegend) [26]. After incubation for 15 min. at room temperature, cells were analyzed by flow cytometry (10,000 events per sample) and data were expressed as mean fluorescence intensity (MFI).

### TLR blockage assay

In order to evaluate whether ATF is able to activate monocyte functions through Toll-like receptors, TLR2 and /or TLR4 were blocked with specific mAb. PBMC were incubated with 0.1 and 0.5 μL of these specific antibodies (DB-Pharmingen) for 1 h and then exposed to ATF (50 μg) for 6 h. After this period, cells were washed and challenged with a *C. albicans*-FITC suspension. Culture supernatants were collected for further analysis of cytokine production.

### Cytokine analysis

PBMC suspension (10^6^ monocytes/mL) was distributed into 24-well tissue culture plates (500 μL) and incubated for 2 h at 37 °C under 5% CO_2_. Non-adherent cells were removed and adherent cells were cultured for 6 h with 50 μg ATF. *Escherichia coli* lipopolysaccharide (LPS) at 5 μg/mL was used as positive control. After incubation, supernatants were collected to analyze the levels of TNF-α, IL-1β, IL-12p70 and IL-10 by sandwich enzyme-linked immunosorbent assay (ELISA), using pairs of monoclonal antibodies (mAb) and recombinant control cytokines purchased from R&D Systems (USA) and BioLegend (USA), according to instructions of the manufacturers.

### Statistical analysis

Paired Student’s *t* test was applied to compare data of two groups (expression of PPRs), while multiple comparisons were made by analysis of variance (ANOVA) followed by Student-Newman-Keuls or Tukey-Kramer analysis (as indicated in the figures). Differences were considered significant when the probability of error was lower than 5% (*p* < 0.05).

## Results

### ATF increases the adherence/phagocytosis of *C. albicans*

**Fig. 1:**
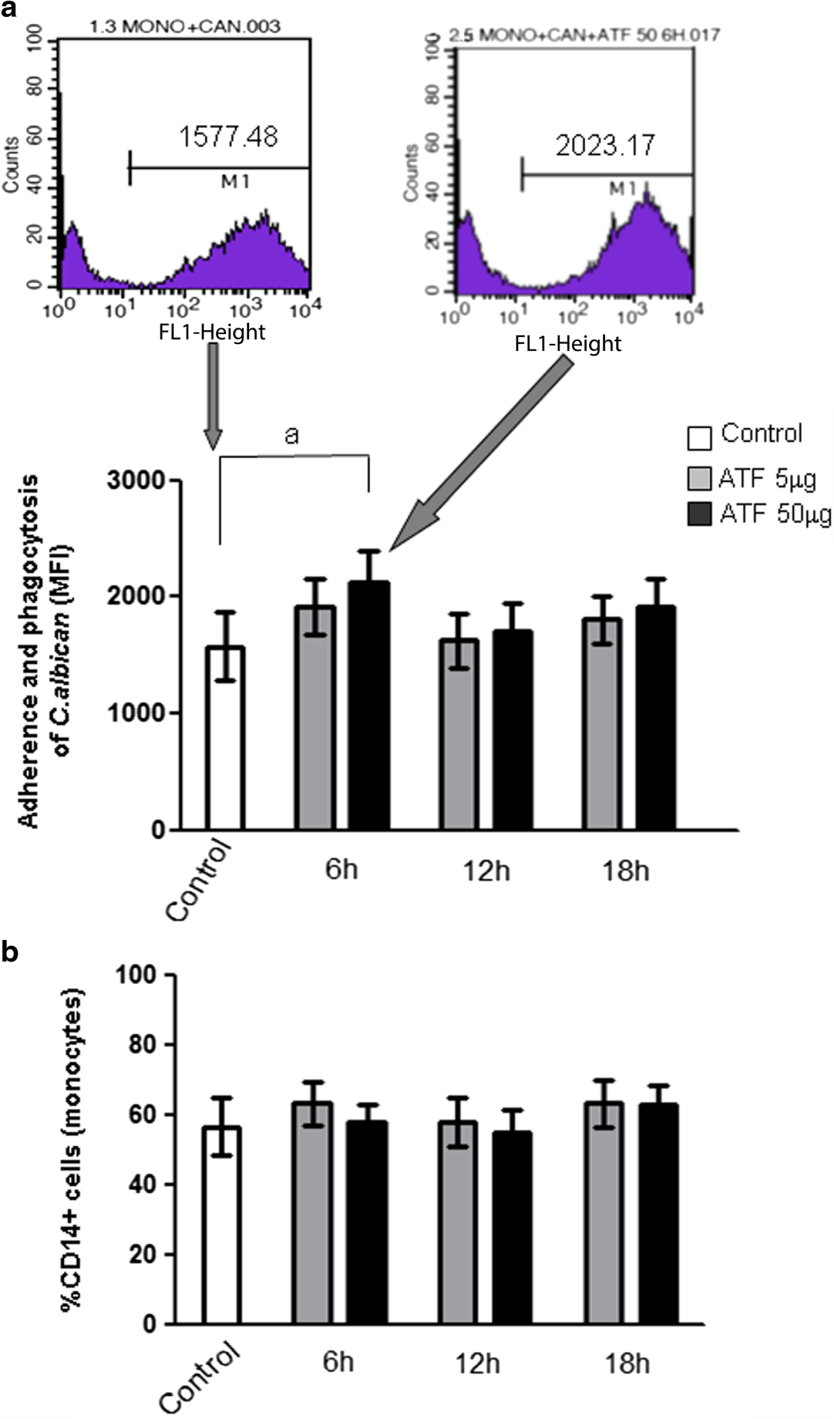
**a** Effect of ATF on the adherence/phagocytosis of *C. albicans-*FITC evaluated by flow cytometry. Results are expressed as mean ± standard deviation (SD) of mean fluorescence intensity (MFI) detected in cells from seven subjects (a: *p* < 0.01) analyzed in two independent experiments. Representative flow cytometry histograms of one out of seven analysis. **b** Effect of ATF on the percentage of CD14^+^ human cells involved in the adherence or phagocytosis of *C. albicans-*FITC determined by flow cytometry. Results are expressed as mean ± SD. Statistical analysis byS Student-Newman-Keuls multiple comparisons test

The ability of ATF to stimulate the phagocytic activity of human monocytes was measured by flow cytometry considering the number of *C. albicans-*FITC that adhered to or was phagocytosed by adherent cells. We observed that the in vitro treatment of cells with 50 μg of ATF for 6 h resulted in an increased number of cells adhering or phagocytosing yeast cells (control = 1574.42 ± 293.47; ATF = 2129.23 ± 266.65; *p* < 0.01 – Fig. 1a). As expected, the relative number of CD14+ cells was not changed by exposition to ATF (Fig. 1b).

### Enhanced phagocytosis of *C. albicans* is associated with increased TLR2 and TLR4 expression

**Fig. 2:**
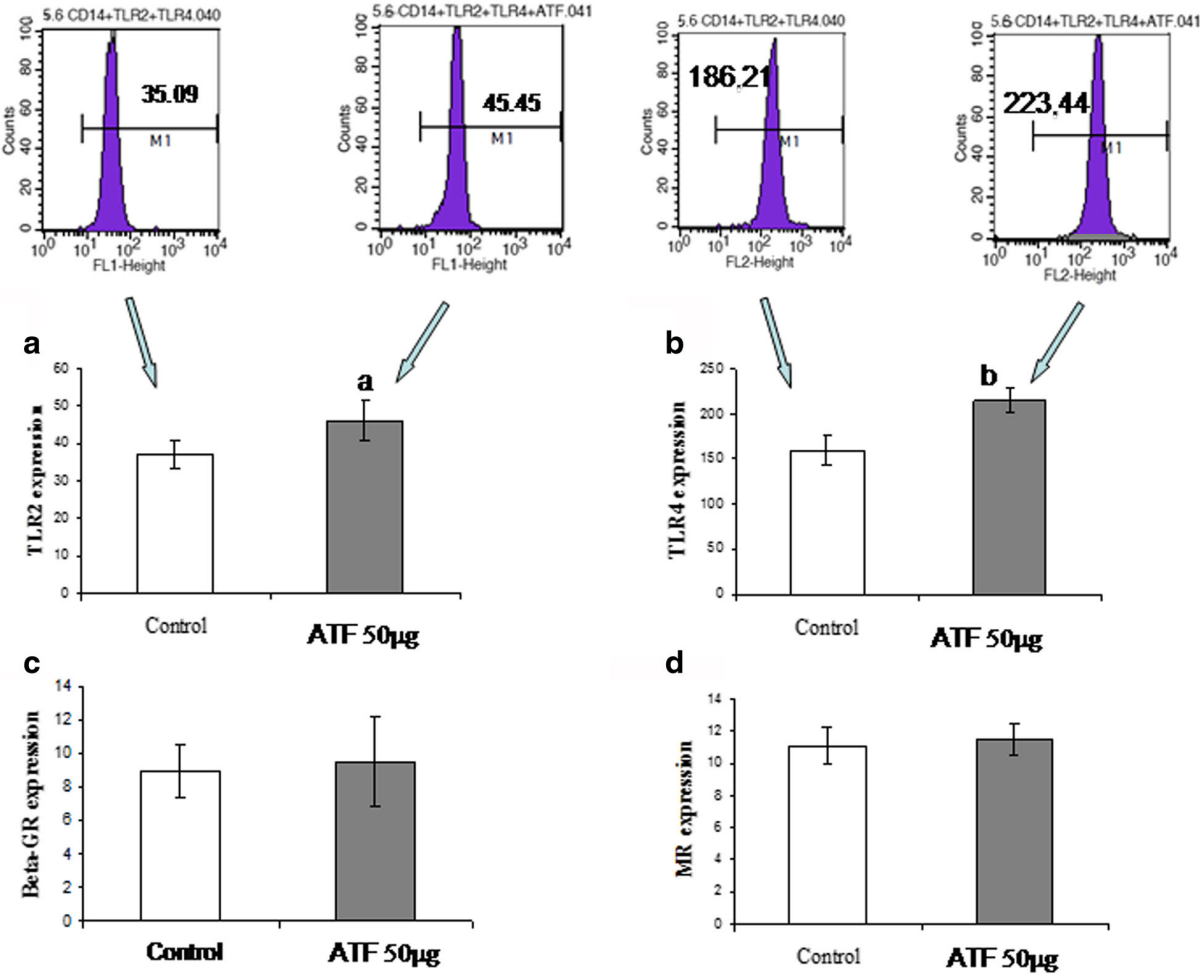
Effect of ATF on the expression of **a** TLR2, **b** TLR4, **c** βGR and **d** MR by human monocytes. Results are expressed as mean ± SD of fluorescence intensity (MFI) detected in cells obtained from seven subjects analyzed in two independent experiments. Statistical analysis by paired Student’s *t* test (a: *p* < 0. 05; b: *p* < 0. 01)

Our results on phagocytosis of yeast cells raised the question of whether the increased adherence/phagocytosis of *C. albicans-*FITC induced by ATF would be due to its ability to modulate some receptors involved in innate immunity. In Fig. 2, we show that the treatment of monocytes with ATF increased the expressions of TLR2 (Fig. 2a) and TLR4 (Fig. 2b) but not βGR (Fig. 2c) or MR (Fig. 2d). Analysis of cells from six different donors showed that the mean fluorescence intensity (MFI) of TLR2 expression increased from 36.99 ± 3.69 to 46.24 ± 5.36 after treatment (*p* < 0.05), whereas TLR4 expression changed from 158.96 ± 16.75 to 214.84 ± 13.93 (*p* < 0.01).

**Fig. 3:**
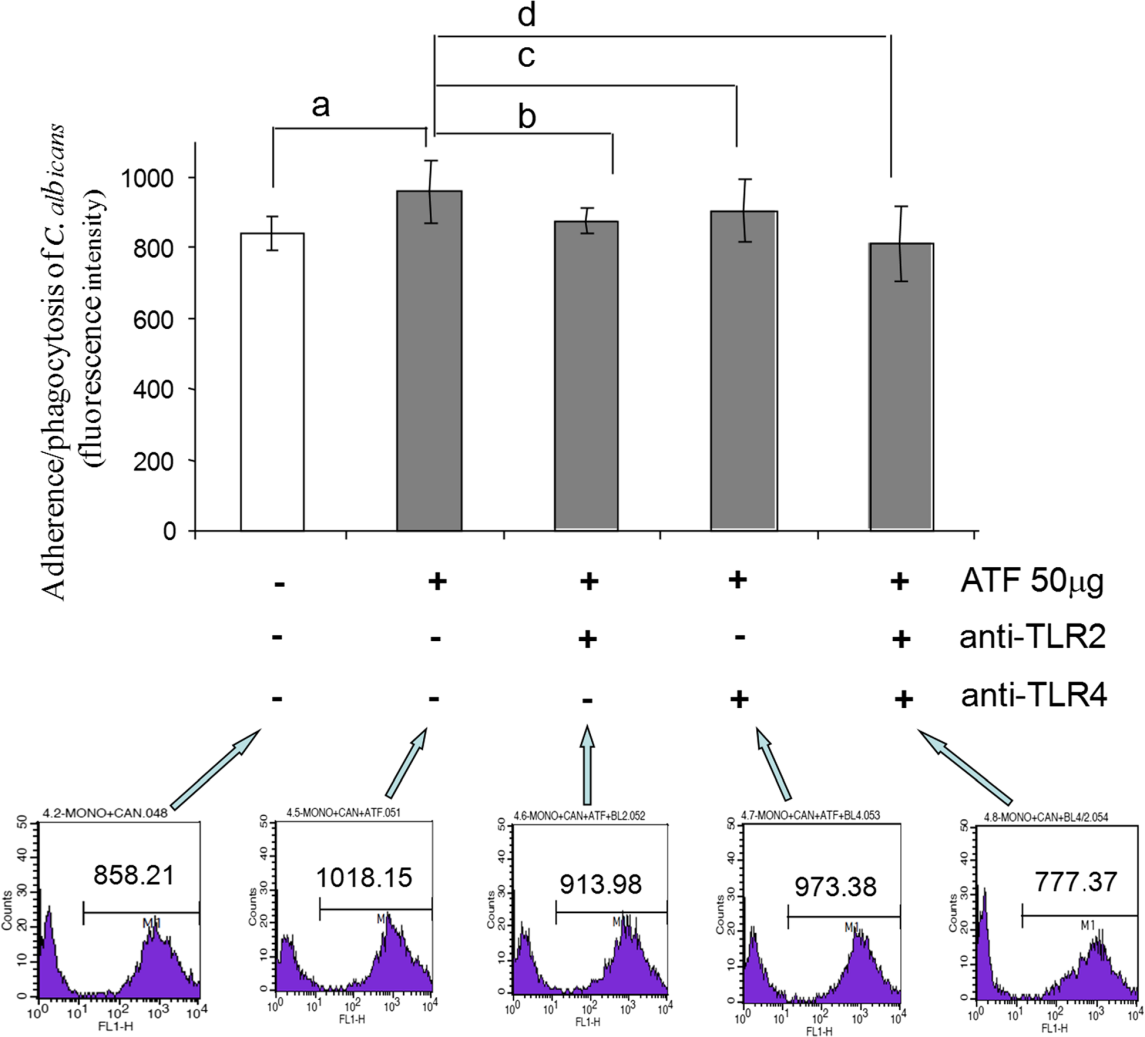
Role of TLR2 and TLR4 in ATF-induced adherence/phagocytosis of *C. albicans*. The results are expressed as mean ± SD of mean fluorescence intensity (MFI) detected by flow cytometry in cells from seven normal subjects analyzed in two independent experiments. Flow cytometry histogram of one representative analysis statistical analysis by Student-Newman-Keuls multiple comparisons test (a: *p* < 0.001; b: *p* < 0.01; c: *p* < 0.05; and d: *p* < 0.001)

Based on these results, we chose the concentration of 50 μg of ATF to treat PBMC for 6 h, in order to evaluate whether the increased adherence/phagocytosis of *C. albicans* was dependent on the TLR2 and TLR4 expression. For this goal, we blocked these receptors with specific mAbs before the adherence/phagocytosis test. Results at Fig. 3 show that blocking TLR2 or TLR4 on cell surface significantly decreased the effect of ATF on adherence/phagocytosis, and this reduction was more evident when both receptors were simultaneously blocked.

### ATF increases the production of cytokines by monocytes

**Fig. 4:**
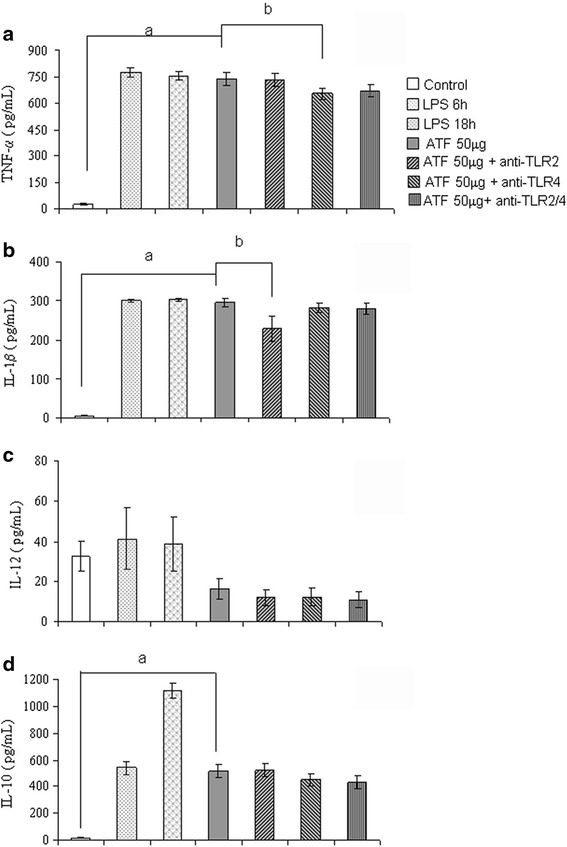
Effect of ATF on the production of **a** TNF-α, **b** IL-1β, **c** IL-12 and **d** IL-10 by human monocytes submitted or not to TLR2 and TLR4 blocking. Results are expressed as mean ± SD of cytokine levels released by cells obtained from 15 normal subjects, which supernatants were obtained in three independent experiments. Statistical analysis by Tukey-Kramer multiple comparisons test (a: *p* < 0.001; b: *p* < 0. 05)

Our next step was to evaluate whether the modulation of TLR2 and TLR4 expression induced by ATF would be associated with changes in cytokine production by monocytes (Fig. 4). We observed that ATF stimulates the production of both TNF-α and IL-1, similarly to LPS used as positive control (Fig. 4a). In addition, we aimed to evaluate if this profile would be dependent on TLR activation. We observed that production of TNF was significantly decreased when TLR4 was blocked by specific antibody, whereas the TLR2 blockage significantly inhibited IL-1β production (Fig. 4b). It is interesting to note that IL-10 secretion was induced by ATF but it was not affected by TLR2 or TLR4 blockage (Fig. 4d). In contrast, ATF tended to decrease the production of IL-12p70, but no significant difference was observed and non-treated monocytes produced very low levels of this cytokine as well (Fig. 4c).

As stated in the methods section, contaminating endotoxins in ATF were measured by *Lymulus amebocyte* lysate test. We found a concentration of only 0.06 EU/mL of endotoxin, which proved that our results were not due to a role of unspecific cytokine stimulator.

## Discussion

In the present study we evaluated the effect of a polysaccharide-rich fraction (ATF) obtained from the mushroom *A. brasiliensis* on the phagocytic activity of human monocytes and the role of PRR in its modulation. As we previously described, ATF has 3.7192 mg/mL (13.4%) of protein, and sugar corresponds to 86.6% of its content [1]. The ^13^C NMR spectrum has shown six well-shaped peaks indicating that the ATF compound is a homogeneous polymer. The anomeric hydrogens were detected at δ 4.49, which was confirmed by the gHSQC spectrum showing correlation of ^1^H at δ 4.49 with ^13^C at δ 103.4, with C-1 β configuration [1].

First, we observed that ATF increases the ability of monocytes to adhere to or phagocytose *C. albicans*. Even though modest, this effect was statistically significant by 6 h of incubation, decreasing in the subsequent time points. Normalization of this reactivity after 12 and 18 h was not expected. In fact, such interaction should have improved the ability of monocytes to phagocytose yeast cells as a response for PRRs. Therefore, since data are expressed as MFI, we could only suggest that early phagocytosed particles were rapidly digested by monocytes, reducing the bright intensity 12 and 18 h later.

We also observed that polysaccharide fraction significantly increased the expression of TLR2 and TLR4. Moreover, experiments blocking these receptors gave us evidence that they mediated the adherence/phagocytosis *of C. albicans* to monocytes, since such blockage decreased the number of adhered/phagocytosed yeast cells to the same levels of untreated control. It is reinforced by the observation that no effect was observed on the percentage of cells involved in this activity, that is, the same number of cells interacted with higher number of yeast cells after treatment with ATF. Since some monocytes submitted to the blocking treatments were still able to interact with yeast cells, we think that these cells are other PRRs for fungal recognition.

Our observation that ATF can modulate TLR2 and −4 is supported by other studies showing their modulation by substances derived from mushrooms and plants. For instance, the extract ABH from *A. blazei* containing β-glucans induces the production of IL-12 by human monocytes by modulating TLR4 expression [27]. Polysaccharides obtained from the mushroom *Phellinus linteus* were shown to increase the functional and phenotypic maturation of dendritic cells through TLR2 and TLR4 modulation [28]. Likewise, polysaccharides extracted from the root of *Platycodon grandiflorum* can activate macrophages by TLR4/NF-kB modulation, supporting our observations on TLR2 and TLR4 modulation [29].

In spite of its action on TLR expression, ATF had no effect on the expression of βGR or MR. The role of MR in the modulation of phagocytosis of *C. albicans* is controversial, since there are studies suggesting both positive and negative roles of this receptor on *C. albicans* uptake. Although its role on the production of cytokines such as MCP-1 and TNF-α is well established, we observed no relationship between MR and TNF-α production [30, 31]. We also have previously demonstrated that treatment of mice with ATF increases phagocytosis and killing of *C. albicans* by murine macrophages, and this effect was associated with higher expression of MR [15]. One possible explanation for this difference is that monocytes used in this study and macrophages used in the murine investigation represent two different stages of cell differentiation and, therefore, can show different behavior after interaction with different ATF components. This hypothesis is supported, for instance, by Ferwerda et al. [32], who demonstrated that macrophages express higher MR levels than monocytes. We may also suggest that PRRs of human and murine cells show differences in reactivity with ATF components, that is, some structures that react with murine PRR could not be recognized by human receptor. It happens, for instance, with some CpG oligonucleotides that react with murine TLRs and are not recognized by human homologous receptors, suggesting a certain degree of selectivity of this PRR [33].

Lack of effect on βGR expression was also not expected in our study since dectin-1 is the main receptor involved in the uptake of *C. albicans* and βGR is the human homolog of murine dectin-1 [20, 21, 30, 34]. This receptor also provides signals for the production of reactive oxygen intermediates (ROI) and for the release of cytokines such as TNF-α, IL-2, IL-6, IL-10, and IL-23 [35–37]. In addition, Ohno’s group have demonstrated that immunoenhancing effects of *A. brasiliensis-*derived β-glucans are mediated by the dectin-1-signaling pathway [38]. However, as reported by this same research group, the amount of β-glucan in ATF can be not enough to efficiently activate βGR [39]. Therefore, in the present study the effect of ATF on cytokine production seems to be more closely associated with its interaction with TLRs than with βGR.

Increased TNF-α and IL-1 production by human monocytes is in agreement with their higher ability to adhere to or phagocyte *C. albicans*, and with the higher candidacidal activity we previously observed in the murine model [15]. Our data are supported by the report of Sorimachi et al. [9] showing that an aqueous extracts of *Agaricus blazei* significantly increased the TNF-α and IL-8 production by bone marrow-derived murine macrophages [9]. Oral administration of another *Agaricus* extract induces murine peritoneal macrophages to release higher IL-1β and IL-6 levels, while human mononuclear cells produce IL-8, IL-6, TNF-α and IL-1β, but not IL-12 [40, 41].

We also observed that the increased TNF-α levels were associated with TLR4 expression, contrasting with the findings of Netea et al. [42], who suggested that TLR4 is not involved in TNF-α, IL-1-α or IL-1β production. Despite this divergence, their results support our suggestion that there is an association between IL-1β production and TLR2 expression. This result is also in agreement with Yamanaka et al. [39], who reported that a water extract of *A. brasiliensis* is able to induce the production of several cytokines by spleen cells being the IL-6 production dependent on TLR-2 activation.

The effect of TLR2 on cytokine production and resistance to infectious diseases has been a controversial point in the literature. In fact, some studies showed that TLR2-defective mice infected with *C. albicans* are less resistant to infection, due to their inability to release TNF-α and MIP-2 or TNF-α, IL-12 and IFN-γ [43, 44]. In opposition, other groups have reported that TLR2-defective mice are more resistant to infection due to increased chemotaxis and candidacidal capacity of macrophages [42]. It is also suggested that the importance of TLRs for host resistance against *C. albicans* is not related to their ability to modulate the release of pro-inflammatory cytokines, but rather to the release of chemokines such as KC and MIP-2.

Curiously, ATF also induced a significant increase of IL-10 production, but it was not associated with either TLR2 or TLR4 expression. This finding is in agreement with our previous observation that ATF increases the production of IL-10 when inoculated in normal mice [1]. This observation is coherent with the tendency of reduced production of IL-12, but contrasts with data reported by Kasai et al. [27] who have observed that β-glucans from *A. blazei* are able to induce IL-12 production by murine macrophages. Again, it is possible that human and murine cells have different response to β-glucans. The balance between pro- and anti-inflammatory responses is essential for successful host–fungal interactions [45]. Although inflammation is crucial for a protective response to fungi, subsequent anti-inflammatory signals (for instance, IL-10) are required to protect the host against the deleterious effects of an overwhelming response after the elimination of an invading microorganism [46].

Taken together, our data on cytokine production strongly suggest that ATF is able to modulate the host response by activating both pro- and anti-inflammatory mechanisms thus increasing the production of TNF-α and IL-1 by human monocytes through modulation of TLR4 and TLR2 expression. These pro-inflammatory cytokines seem to improve the activation of monocytes and increase their ability to phagocyte *C. albicans* yeast cells. Since monocytes still produce considerable levels of IFN-γ, IL-1β and IL-10, even after the TLR blockade, we speculate that ATF also activate these cells by TLR-independent mechanisms.

## Conclusions

Our results provided us with the evidence that *A. brasiliensis* polysaccharides increase the activity of human monocytes, probably through the modulation of Toll-like receptors TLR4 and TLR2, and the increased production of TNF-α and IL-1β.

## Abbreviations

ATF: Acid-treated fraction
ELISA: Enzyme-linked immunosorbent assay
FITC: Fluorescein isothiocyanate
LPS: *Escherichia coli* lipopolysaccharide
mAb: Monoclonal antibody
MFI: Medium fluorescence intensity
MR: Mannose receptor
PAMP: Pathogen-associated molecular pattern
PBMC: Peripheral blood mononuclear cell
PRR: Pattern recognition receptor
ROI: Reactive oxygen intermediates
TLR: Toll-like receptor
βGR: β-glucan receptor
